# Intramuscular vs. Subcutaneous: Rethinking Influenza Vaccination Strategy in Japan

**DOI:** 10.31662/jmaj.2023-0122

**Published:** 2023-12-11

**Authors:** Yudai Kaneda, Uiri Kaneda, Akihiko Ozaki, Tetsuya Tanimoto

**Affiliations:** 1 School of Medicine, Hokkaido University, Hokkaido, Japan; 2 Faculty of Foreign Languages, Dokkyo University, Saitama, Japan; 3 Department of Breast and Thyroid Surgery, Jyoban Hospital of Tokiwa Foundation, Fukushima, Japan; 4 Department of Internal Medicine, Jyoban Hospital of Tokiwa Foundation, Fukushima, Japan

**Keywords:** Subcutaneous Vaccination, Influenza, COVID-19, International Standards, Japan

## Abstract

In Japan, inactivated vaccines, including the influenza vaccine, are administered subcutaneously, which is contrary to global recommendations for intramuscular injections. This practice is attributed to historical medical incidents and unchallenged conventions. However, this outdated method, which differs from that of international standards and is linked with less immunogenicity and more adverse reactions, may contribute to vaccination hesitancy. Therefore, with the adoption of intramuscular vaccination administration, which was widely adopted in the coronavirus disease 2019 vaccination, a shift in the Japanese health policy to conform to international standards potentially improves vaccine acceptance and effectiveness.

Since the outbreak of the coronavirus disease 2019 (COVID-19), a decline in the number of influenza cases has been reported in many countries worldwide between March 2020 and September 2021, including Japan, where the number of cases has decreased by more than 99% compared with that of the prepandemic period ^(1)^. This could be attributed to compliance with health principles such as social distancing and the use of masks, which were implemented as a COVID-19 countermeasure. Before the COVID-19 pandemic, the annual influenza incidence rates in Japan were estimated at 55 cases per 1000 person-years, and about 80% of school-aged children and 50% of the elderly were vaccinated yearly ^(1)^. Vaccination has been reported to reduce the risk of infection by between 40% and 60% among the overall population during seasons when most circulating influenza viruses are well matched to those in the influenza vaccines ^(2)^. Beyond the health benefits, from socioeconomic perspectives, influenza vaccination has also been cited as a cost-effective strategy ^(3), (4)^; for example, it has been reported that vaccination results in a 23% reduction in absenteeism and a 30% decrease in lost workdays, underscoring that the benefits of vaccination outweigh the associated costs ^(5)^. However, based on data from the 2022 and 2023 winter seasons, the number of influenza cases saw a sharp resurgence in the northern hemisphere, with Japan witnessing a staggering increase where the reported cases in September 2023 are 166 times higher than those of the previous year ^(6)^. In this context, since there are no definitive clinical symptoms that allow physicians to distinguish COVID-19 from influenza ^(7)^, vaccination for prevention becomes increasingly important.

The newly introduced imported mRNA COVID-19 vaccine made by Pfizer and Moderna, which are mainly used in Japan, is administered intramuscularly as in other countries ^(8)^. In contrast, the inactivated influenza vaccine has been administered subcutaneously for decades. This is partially due to Japan’s health policy, which has so far produced all of its influenza vaccines domestically by several Japanese companies and has rarely imported them from other countries. Regardless of the vaccine type, it has been reported that intramuscular vaccine administration consistently demonstrates superior immunogenicity over subcutaneous vaccine administration, evidenced by fewer local adverse reactions, enhanced antibody responses, and favorable outcomes regarding protection rates and antibody titers ^(9)^. Specifically, although studies specific to the influenza vaccine are limited, it has been reported that the incidence rate is significantly lower at 8.2% for intramuscular injections compared with 11.3% for subcutaneous injections, with intramuscular injections also yielding significantly more favorable results in terms of pain during vaccination and subsequent adverse reactions ^(10)^. Indeed, the Centers for Disease Control and Prevention in the U.S. and the World Health Organization recommend intramuscular injection for inactivated vaccines, including influenza ^(11), (12)^. However, contrary to the international standard, in Japan, many inactivated vaccines, including the influenza vaccine, have been, for decades, administered subcutaneously.

The subcutaneous administration of inactivated vaccines in Japan is due to a social problem in the 1970s that resulted in tens of thousands of cases of quadriceps muscle contracture caused by the intramuscular injection of antipyretic drugs and antibiotics for children ^(13)^. This led to class action lawsuits and the avoidance of intramuscular injections in children throughout various medical practices, which also resulted in the avoidance of the intramuscular injection of vaccines. Considering the situation at that time, such medical practice would have been inevitable. However, the conventional practice of subcutaneous influenza vaccine inoculation has remained unconsidered for nearly half a century, although tens of millions of Japanese people routinely receive subcutaneous injections of the influenza vaccine and other inactivated vaccines every year.

Revising the vaccine administration method requires a certain cost for the company and the government because they usually need to conduct registered clinical trials to prove the safety and efficacy and apply for official regulatory approval. In addition, in the case of an adverse reaction, no excuses are allowed as the injection method differed from that in the attachment. Therefore, there was no incentive to change to the intramuscular route of vaccine administration as subcutaneous vaccine administration of the influenza vaccine has already been widely accepted in Japan without any major inconveniences. Moreover, there may have been an unnecessary status quo bias, assuming that the change would confuse the medical field.

However, with the recent nationwide adoption of the COVID-19 vaccine in Japan, intramuscular vaccine administration has become prevalent among medical practitioners, and severe adverse reaction rates have been reported to be <0.001% ^(8)^. On the contrary, it has been reported that subcutaneous vaccine administration is an outdated method that is less immunogenic and has more adverse reactions, leading to vaccine hesitancy in the population ^(9)^. Of note, as a policy shift has been recently under discussion toward introducing not only domestically produced influenza vaccines but also imported foreign vaccines ^(14)^, the coexistence of multiple vaccine administration methods may confuse the medical field.

In Japan, vaccine approval is governed by the Ministry of Health, Labor, and Welfare. While there are inherent challenges, such as adherence to the traditional principle of infallibility ^(15)^, the current influenza outbreaks offer a pivotal moment for reflection on the subcutaneous administration of inactivated influenza vaccines within the country. Drawing parallels with the process adopted for changes in pediatric dosages and frequency of influenza vaccines in Japan, a systematic progression must be followed: starting from shifting public opinion, then guiding administrative decisions, collaborating with domestic pharmaceutical consortiums for trials, and culminating in the revision of official guidelines ^(16)^. In this process, the capacity to offer appropriate incentives to the government and corporations in terms of effort and budget is crucial for safeguarding public health, emphasizing the importance of shifting public opinion through the dissemination of accurate information.

In conclusion, as Japan grapples with its first influenza outbreak in three years after the COVID-19 pandemic ^(6)^, it is evident that the nation stands at a pivotal juncture. While both domestically produced and imported vaccines demonstrate equivalent efficacy ^(17)^, the longstanding practice of the subcutaneous administration of the influenza vaccine in Japan deviates from global standards. Considering the demonstrated benefits of intramuscular vaccine administration, such as enhanced immunogenicity and fewer adverse reactions ^(9)^, and the recent widespread acceptance of intramuscular COVID-19 vaccinations in the country ^(8)^, a policy shift is both timely and crucial. By aligning with the international health standards for intramuscular administration or considering intradermal administration, which has been reported to possess equivalent efficacy to subcutaneous administration ^(18)^, Japan has the potential to not only enhance public health but also reduce vaccine hesitancy and ensure a more effective response to future health challenges.

## Article Information

### Conflicts of Interest

Dr. Ozaki reported personal fees from Medical Network Systems Inc. and Kyowa Kirin Co. Ltd. outside the submitted work. Dr. Tanimoto reported personal fees from Medical Network Systems Inc. and Bionics Co. Ltd. outside the submitted work.

### Author Contributions

Conception and design of the study: YK and TT

Writing of the original draft: YK and UK

Critical revision of the paper: AO and TT

All the authors read the final draft and approved the submission.
